# Hemangiopericytoma in a young dog: Evaluation of histopathological and immunohistochemical features

**Published:** 2014

**Authors:** Fatemeh Namazi, Mohammad Abbaszadeh Hasiri, Ahmad Oryan, Ali Moshiri

**Affiliations:** 1 *Department of Pathobiology, School of Veterinary Medicine, Shiraz University, Shiraz, Iran;*; 2 *Department of Clinical Studies, School of Veterinary Medicine, Shiraz University, Shiraz, Iran.*

**Keywords:** Dog, Hemangiopericytoma, Histopathology, Immunohistochemistry

## Abstract

In the present study, we describe a subcutaneous mass between the left flank and hip in a 2-year-old male Great Dane dog. Histopathologically, cells appeared to be spindle shaped around a central capillary together with a fingerprint pattern. Immunohistochemical analysis presented that the neoplastic cells expressed vimentin, but did not stain for S-100 protein. On the basis of histopathology and immunohistochemical findings, the present tumor was diagnosed as canine hemangiopericytoma. Hemangiopericytoma could be considered in differential diagnosis list of any mass in the skin (even in young dogs) and must be identified histopathologically.

## Introduction

Hemangiopericytoma was first described in human by Stout *et al*. and recognized in dogs 4 years later.^1^^,^^2^ This neoplasm is a spindle cell tumor, arising in subcutis, which is common in dogs and rare in cats. The neoplastic cells surround capillaries and post-capillary venules. This mesenchymal neoplasm derives from vascular pericytic contractile cells around vessels.^3^^,^^4^ In humans, hemangiopericytomas are often malignant and can involve many structures such as central nervous system, viscera, and somatic soft tissues.^5^ Most of these tumors develop in deep soft tissues.^6^ However, canine hemangiopericytomas are almost often found in subcutaneous layer of integument of the extremities and are classified as malignant connective tissue tumors.^7^ Recently, the epitheloid, storiform and perivascular forms have been described as morphological subtypes of hemangiopericytoma.^8^ It has been shown that epithelioid form is the most common and aggressive subtype.^9^

Hemangiopericytomas are typically diagnosed on middle-aged or older dogs (average age is 7 to 10 years). The large breeds of dogs appear over-represented, but there is no significant sex predilection.^4^^,^^10^ The best recommended treatment for hemangiopericytoma is to surgical removal of the mass with wide margins. If the total lump and a substantial healthy rim surrounding the neoplastic mass are removed, re-occurrence of the growth is unlikely, and it has been stated that approximately 70% of these neoplasms can be controlled by surgical excision.^3^ When the tumor recurs, it becomes more aggressive;^11^^,^^12^ however, they rarely metastasize in dogs.^13^ Histopathological analysis together with classification of subtypes, quantification of cell proliferation and apoptosis rates have been reported helpful to determine prognosis of this tumor.^9^

**Case Description**

**Fig. 1:**
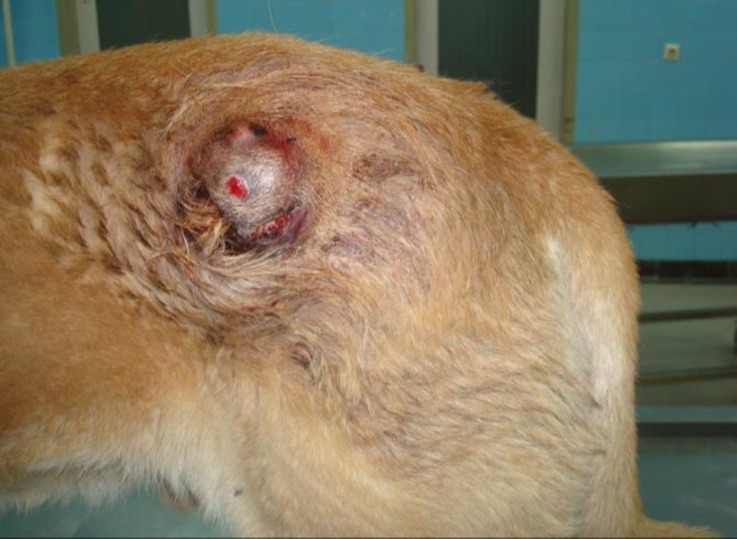
Gross appearance of the subcutaneous mass. The skin over the neoplasm was alopecic and ulcerated

A 2-year-old male Great Dane dog was evaluated for a cutaneous mass. This mass was located between the left flank and hip, raised in subcutis and it was approximately 5 cm in diameter. The skin over the neoplasm was alopecic and ulcerated (Fig. 1). Complete blood count, thoracic radiographs and popliteal lymph node size were normal. The mass was removed by excisional biopsy. The sample was fixed in 10% neutral buffered formalin and sections of the tumor were stained with hematoxylin and eosin (H & E) for histopathological evaluation. In addition, an immunohistological analysis was performed to differentiate the tumor from peripheral nerve sheath tumor and confirm the histopathological diagnosis. Immunohistochemical expression of vimentin and S-100 protein were used in formalin-fixed, paraffin-embedded sample and sections of 5-µm in thickness were processed with avidin-biotin-peroxidase complex (ABC) technique. Mayer’s hematoxylin was used for counter staining. 

## Results

**Fig. 2:**
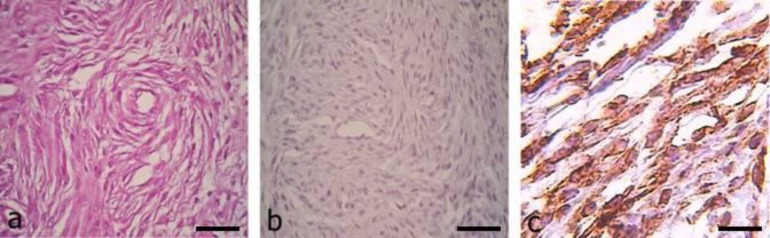
Canine hemangiopericytoma, subcutis. **a)** Spindle shaped cells are arranged around a central capillary, a characteristic "fingerprint pattern" appearance, in the collagenous stroma is seen. H & E. Bar =56 µm; **b)** As it is expected immunolabelling with S-100 is negative for this canine hemangiopericytoma. Bar =56 µm; **c)** Neoplastic cells are positive by immunolabelling with vimentin. Bar =56 µm

Grossly, the mass was a solitary, circumscribed, greyish-white and demonstrated a firm consistency. Histopathological features revealed a hypercellular pattern similar a fingerprint. On a higher magnification, the individual cells appeared to be multiple layers of spindle shaped around a central capillary, forming whorls, together with collagenous stroma. The predominant cells had eosinophilic cytoplasm with prominent nuclei. The mitotic figures were scarce (Fig. 2a). 

By application immunohistochemical staining, the tumor cells expressed vimentin, but did not stain for S-100 protein (Figs. 2b and 2c). On the basis of the histopathological and immunohistochemical findings, the tumor was diagnosed a subcutaneous canine hemangiopericytoma. Recurrence or other masses on the skin were not seen in 6 month follow up.

## Discussion

Some neoplasms have exclusive histopathological features and can be distinguished from other tumors by pathological analyses. On histopathological evaluation of the present case, a fingerprint pattern around a central capillary was seen as the hallmark of hemangiopericytoma and consequently the perivascular subtype of hemangiopericytoma was diagnosed. Because occasional cases of soft tissue tumors may present the fingerprint pattern, differential diagnosis of these tumors without immunohistochemical analyses is often impossible, so various techniques such as immunohistochemical and ultrastructural studies have been used to diagnose and evaluate hemangiopericytomas.^7^^,^^14^ In addition, recent studies indicate that hemangiopericytomas have been over-diagnosed in both humans and dogs, as the diagnostic term “hemangiopericytoma” is often used to denote the histologic pattern created by a variety of spindle cell tumors with a whorling pattern, rather than a specific tumor of pericytes.^13^^,^^15^^,^^16^ The ABC staining technique has been used for evaluation of nuclear and cytoplasmic activation in hemangiopericytoma. Hemangiopericytomas may appear histologically similar to peripheral nerve sheath tumors including schwannomas and neuroﬁbromas, ﬁbrosarcoma or synovial sarcoma and thus they should be differentiated from each other. In contrast to hemangiopericytoma, whorls in peripheral nerve sheath tumor are less noticeable, and most of them surround sclerotic collagen rather than capillaries. In addition, focal spindle cell areas are rarely observed in the sections of hemangiopericytoma, but these cells are never arranged in long bundles or fascicles as in ﬁbrosarcoma or synovial sarcoma.^17^ S-100 protein is a valuable immunohistochemical marker for identification of neural differentiation^18^ which peripheral nerve sheath tumors are generally positive for vimentin and S-100^19^. In the present study, the lack of staining for S-100 protein for hemangiopericytoma distinguishes it from peripheral nerve sheath tumor and supports a former diagnosis of canine hemangiopericytoma. Negative immunohistochemical stain for S-100 protein has also been found in human hemangiopericytomas.^20^^,^^21^

Hemangiopericytoma could be considered in differential diagnosis list of any mass in the skin especially on limbs. Although this tumor has been reported more in aged dogs, our case showed it could happen on young dogs too. Any subcutaneous mass, in any age, must be identified histopathologically and in suspicion of tumor removed completely. 

## References

[1] Stout AP, Murray M, Gabbiani R (1942). Hemangiopericytoma: A vascular tumor featuring Zimmerman’s pericytes. Ann Surg.

[2] Pulley LT, Stannard AA, Pulley LT, Stannard AA, Moulton JE (1990). Tumors of the skin and soft tissue. Tumors in domestic animals.

[3] Mazzei M, Milanta F, Citi S (2002). Hemangiopericytoma: Histological spectrum, immunohistochemical characterization and prognosis. Vet Dermatol.

[4] Meuten DJ (2002). Tumors in domestic animals.

[5] Madewell BR, Griffey SM, Munn RJ (1992). Ultrastructure of canine vasoformative tumors. J Vasc Res.

[6] Miyauchi A, Fukase M, Tsutsumi M (1988). Hemangiopericytoma-induced osteomalacia: Tumor transplantation in nude mice causes hypophosphatemia and tumor extracts inhibit renal 25-hydroxyvitamin D 1-hydroxylase activity. J Clin Endocrinol Metab.

[7] Nappi O, Ritter JH, Pettinato G (1995). Hemangiopericytoma: Histological pattern or clinical entity?. Semin Diagn Pathol.

[8] Goldschmidt MH, Henderick MJ, Meuten DJ (2002). Tumors of the skin and soft tissue. Tumors of domestic animals.

[9] Santos SV, Torres LN, da Silva TC (2009). Canine hemangiopericytoma: Cell proliferation and apoptosis in the perivascular, storiform and epithelioid histo-logical subtypes and their significance for prognosis. Braz J Vet Pathol.

[10] Graves GM, Bjorlin DE, Mahaffey E (1988). Canine hemangiopericytoma: 23 cases (1967–1984). J Am Vet Med Assoc.

[11] Richardson RC, Render JA, Rudd RG (1983). Metastatic canine hemangiopericytoma. J Am Vet Med Assoc.

[12] Goldschmidt MH, Shofer OFS, Gold-Schmidt MH (1992). Canine hemangiopericytoma. Skin tumors of the dog and cat.

[13] Perez J, Bautista MJ, Rollon E (1996). Immunohistochemical characterization of hemangiopericytomas and other spindle cell tumors in the dog. Vet Pathol.

[14] Schurch W, Skalli O, Lagace R (1990). Intermediate filament proteins and actin isoforms as markers for soft tissue tumor differentiation and origin Hemangiopericytomas and glomus tumors. Am J Pathol.

[15] Weiss SW, Goldblum JR (2001). Enzinger and Weiss’s soft tissue tumors.

[16] Williamson MM, Middleton DJ (1998). Cutaneous soft tissue tumors in dogs: Classification, differentiation, and histogenesis. Vet Dermatol.

[17] Enzinger FM, Weiss SW (1995). Soft tissue tumors.

[18] Handharyani E, Ochiai K, Kadosawa T (1999). Canine hemangiopericytoma: An evaluation of metastatic potential. J Vet Diagn Invest.

[19] Chijiwa K, Uchida K, Tateyama S (2004). Immunohisto-chemical evaluation of canine peripheral nerve sheath tumors and other soft tissue sarcomas. Vet Pathol.

[20] Nielsen GP, Dickersin GR, Provenzal JM (1995). Lipomatous hemangiopericytoma: A histologic, ultrastructural and immunohistochemical study of a unique variant of hemangiopericytoma. Am J Surg Pathol.

[21] Nakagawa T, Shinoda Y, Masuko Y (1997). Hemangio-pericytoma of the sigmoid mesentery: Report of a case with immunohistochemical ﬁndings. Surg Today.

